# Real-Time PCR Method Combined with a Matrix Lysis Procedure for the Quantification of *Listeria monocytogenes* in Meat Products

**DOI:** 10.3390/foods10040735

**Published:** 2021-03-30

**Authors:** Mirian Labrador, Carlota Giménez-Rota, Carmen Rota

**Affiliations:** 1 Departamento de Producción Animal y Ciencia de los Alimentos, Facultad de Veterinaria, Instituto Agroalimentario de Aragón -IA2-, Universidad de Zaragoza-CITA, C/Miguel Servet 177, 50013 Zaragoza, Spain; miriamlabb@gmail.com; 2 Facultad de Farmacia, Universidad Complutense de Madrid, Plaza Ramón y Cajal s/n, 28040 Madrid, Spain; carlotagimenezrota@gmail.com

**Keywords:** real time PCR, quantification, *Listeria monocytogenes*, meat products, matrix lysis

## Abstract

In this study a real-time PCR method has been developed for the specific quantification of the foodborne pathogen *Listeria monocytogenes* on meat products through the gene *hlyA*. The PCR was combined with a matrix lysis that allowed the obtaining of the microorganisms without sample dilution and the elimination the PCR inhibitors from dry-cured ham. The qPCR method calibration curve had an efficiency of 100.4%, limits of detection and quantification were 30.1 ± 6.2 CFU/g which is under the legal limit of *L. monocytogenes* in ready-to-eat products, and an analytical variability <0.25 log *hlyA* gene copies/reaction. The analysis was performed simultaneously with the reference method ISO 11290-2. The comparison of the qPCR-matrix lysis results with the reference method showed an excellent correspondence, with a relative accuracy between 95.83–105.20%. Finally, the method was applied to commercial derived meat samples and the pathogen was quantified in one of the commercial samples assayed in 69.1 ± 13.9 CFU/g while the reference method did not quantify it. The optimized qPCR showed higher precision and sensitivity than the reference method at low concentrations of the microorganism in a shorter time. Therefore, qPCR-matrix lysis shows a potential application in the meat industry for *L. monocytogenes* routine control.

## 1. Introduction

Nowadays, Spain produces a large number of ready-to-eat pork products such as cold cuts, cured or enriched with flour and spices for its nationwide consume or exportation to third-world countries because of its variety, quality and distinguished and valued taste. Consequently, the pork meat industry implies an important contribution to national economy, positioning Spain as 2nd pork meat producer in the EU after Germany, and 4th producer of elaborated meat products in 2018 (Asociación Nacional de industrias de la Carne de España).

Spanish ready to eat (RTE) meat products security is a continuous challenge to the food industry. Effective control of the different food pathogens is essential, and *Listeria monocytogenes* is one of the most important for public health. This microorganism causes a severe foodborne disease known as listeriosis, which mainly affects certain high risk groups such as pregnant women, infants, elderly, or individuals with a weakened immune system, and has a lethality of 20–30% [1,2]. Besides, *L. monocytogenes* is an ubiquitous organism commonly present on the surface of the food processing machinery, that can also produce biofilms leading to its and the crossed contamination among different products of the production chain [3]. Thus, this microorganism survives and reproduces under unfavorable conditions; low water activity, wide pH range, high concentrations of NaCl, or presence of antimicrobials. Among all, RTE meat products are the one of the main high-risk foods because their exposure to the elaboration surfaces during several steps such as deboning, cut, slicing, or packaging, their relative long shelf-life under refrigeration and its consumption without any treatment that may eliminate the pathogen [4].

Currently, the prevalence of *L. monocytogenes* in meat products in Spain is variable, between 1% and 29.7% depending on the hygienic conditions during its processing [5,6,7]. The European Food Safety Authority has reported a prevalence of this microorganism of 1.3% in pork-derived RTE meat products [8]. Nevertheless, recently in 2019, there was the most important outbreak of *L. monocytogenes* in Spain, with more than 200 cases. It was associated to carne *mechada*, a ready-to-eat meat product traditionally consumed in the south of this country **Centro de Coordinación de Alertas y Emergencias Sanitarias,** *Ministerio de Sanidad, Consumo y Bienestar Social* of Spain)The genetic correspondence of *L. monocytogenes* among the isolated in the food product, industry surfaces, and clinical cases showed that the contamination took place in the industry during the food processing.

In order to avoid the health-related consequences and maintain these products market competitivity, meat industry has to comply with microbiological criteria established by the EU (Commission Regulation (EC) No 2073/2005 (2005) for *L. monocytogenes*. For RTE products that able to support the growth of *L. monocytogenes,* the limit is non-detected *L. monocytogenes* in 25 g of products before they leave the production plant, or 100 CFU/g during its complete shelf-life. For RTE products unable to support the growth of the pathogen, products cannot have greater than 100 CFU/g during their shelf-life [4,8,9].

This Commission Regulation also established the reference methods for the detection and quantification of *L. monocytogenes*, ISO 11290-1 and ISO 11290-2, respectively. Nevertheless, these methods present several limitations such as prolonged procedure, high costs, and has a low sensitivity and precision at low concentrations [10]. Although a low percentage of samples exceeds the legal limit of 100 CFU/g, food industry requires a quick, simple, and efficient method for the detection and quantification of *L. monocytogenes* that can be integrated in its Hazard Analysis and Critical Control Points (HACCP) system.

As alternative to the reference methods, electrochemistry, molecular or immunochemistry techniques have been studied for the quantification of *L. monocytogenes* in food. In the last few years several quantitative PCR methods have been developed because of its advantages in terms of reliability, sensitivity, specificity, automatization, and multiple sample analysis [11,12,13,14,15]. Nevertheless, these techniques require a sample dilution before the analysis in order to reduce the concentration of inhibitors that may affect the PCR reaction efficiency and influence negatively the limit of detection and quantification. On the other hand, the concentration of *L. monocytogenes* in food is usually low, so the dilution of the sample affects the reliable quantification of the pathogen. The analysis of samples without prior dilution is a challenge to address. This implies the proper preparation of the sample to avoid the negative effects of the inhibitors and the concentration of the microorganisms. With this aim, several studies have included different steps such as centrifugation, membrane filtration, or the use of specific buffers [16,17,18]. The new procedures must be validated and compared with the reference method to ensure the results quality.

Because of this, the aim of this work has been the optimization and evaluation of a fast quantitative method based on matrix lysis procedure combined with real time PCR for *L. monocytogenes* in RTE meat products, in order to contribute to the control of this pathogen in meat industry.

## 2. Materials and Methods

### 2.1. Quantitative PCR Optimization

#### 2.1.1. Bacterial Culture Conditions and DNA Extraction

To optimize the PCR amplification conditions, DNA was extracted from a pure culture of *L. monocytogenes* ATCC 19114 (American Type of American Collection). A bacterial colony was grown on 10 mL of Brain Heart Infusion broth (BHI) at 37 °C overnight to a final bacterial concentration of 2–3 × 10^9^ CFU/mL.

DNA was extracted from 1 mL of the overnight culture with DNeasy Blood and Tissue Kit (Qiagen, Hilden, Germany) following the manufacturer’s instructions. Samples were centrifuged at 10,000× *g* for 1 min in a final volume of elution buffer of 150 μL. The final DNA concentration (ng/mL) and its purity was measured by spectrophotometry using Nanodrop 2000 (and the software NanoDrop 2000/2000 c (version 1.3.1) (Thermo Scientific, Waltham, MA, USA). Finally, to perform the PCR analysis, purified DNA was solved in molecular biology grade water.

#### 2.1.2. Quantitative PCR Assay

For the quantification of *L. monocytogenes*, the gene hlyA, a 113 bp fragment, was targeted. Probe and primers set employed in the assays were selected according to a previous study [19]. The reactions contained TaqMan Master Mix 1X (BIORON, Römerberg, Germany), forward primer (125 or 175 nM), reverse primer (125 or 175 nM), Taqman probe (125–250 nM), 5 μL DNA previously extracted (3 pg DNA) and water (final volume 25 μL). The set of primers and Taqman probe are shown in Table 1.

The amplification was performed with Miniopticon^®^ Real Time PCR System (Bio-Rad, Hercules, CA, USA) and the software CFX Manager. The amplification program steps were a pre-incubation at 94 °C for 3 min followed by 45 cycles: denaturalization at 95 °C for 20 s, annealing at 60 to 65 °C for 20 s, and extension at 72 °C for 20 s.

For the optimization of the PCR conditions, all reactions were performed in triplicate and it was included a negative control of the DNA extraction (distilled water), and a positive and negative control of the PCR (*Listeria monocytogenes* DNA ATCC 19114). It was assayed a rage of annealing temperature from 60 °C to 65 °C and different combinations of prime/probe concentrations: 350/250, 350/125, 250/250, and 250/125 nM. A higher final product (end-point fluorescence) and a lower value of Cq (quantification cycle) were the criteria employed to select the annealing temperature and the primer/probe concentrations. A DNA standard curve and a cell standard curve were constructed. For both curves the reaction efficiency (E) was determined according to Equation (1) [20].

(1) $$E\;\left(\%\right)=\left(\left(10^{\;-\;1/slope}\right)\;-\;1\right)\times 100$$

##### DNA Calibration Curve

DNA was extracted from 1 mL of the overnight culture in water were performed to a final range of 3.1 × 10^6^ to 3.1 fg/PCR reaction. Assuming that 3.16 fg of DNA equals the mass of one single whole genome of *L. monocytogenes* and that *hlyA* is present in single copy, the range assayed was from 9.8 × 10^5^ to 0.98 genomic equivalents of *L. monocytogenes*/reaction. Every DNA concentration amplification assay was performed in triplicate in three different days (total assays = 63).

##### Cellular Calibration Curve

From 1 mL of the overnight culture, nine serial tenfold dilutions in peptone water 0.1% were performed to a final range of 2 × 10^7^ to 2 × 10^1^ CFU/mL, approximately. DNA was extracted from each dilution according to Section 2.1.1 and 5 μL of the extracted DNA were analyzed by qPCR in triplicate in 3 different days (total assays = 81). Simultaneously, the bacterial concentration of each dilution was verified by plating on BHI agar (37 °C/24 h).

### 2.2. Quantification of L. monocytogenes in Meat Products

#### 2.2.1. Samples Artificial Contamination

Dry cured ham was selected as meat product to be artificially contaminated. Sample contamination was performed with two aims. First, 30 ham samples of 6 g were divided in 3 sets (set = 10 samples) to be artificially contaminated with serial dilutions of *L. monocytogenes* to evaluate the proposed PCR method linearity, efficiency, precision, analytical variability, and limits of detection (LOD) and quantification (LOQ) (Method A).

Then other 60 ham samples of 6 g each were divided in 6 sets to quantify with the three methods: A, B, and reference ISO 11290-2/A1:2018, and determine the relative accuracy regarding the reference method. A total of 20 samples were analyzed by each method.

To artificially contaminate the ham samples, serial tenfold dilutions from a single *L. monocytogenes* overnight culture broth of 2–3 × 10^9^ CFU/mL were prepared. From each set, nine samples were contaminated with different concentrations of the understudy bacterial strain: 3 × 10^6^, 3 × 10^5^, 3 × 10^4^, 3 × 10^3^, 3 × 10^2^, 3 × 10^1^, 1.5 × 10^1^, 7.5, 3 CFU/g, approximately, and one was used as negative control. Each ham sample was contaminated with 100 μL of the prepared dilutions.

#### 2.2.2. Methods for the Quantification of *L. monocytogenes* in Meat Products

**Figure 1:**
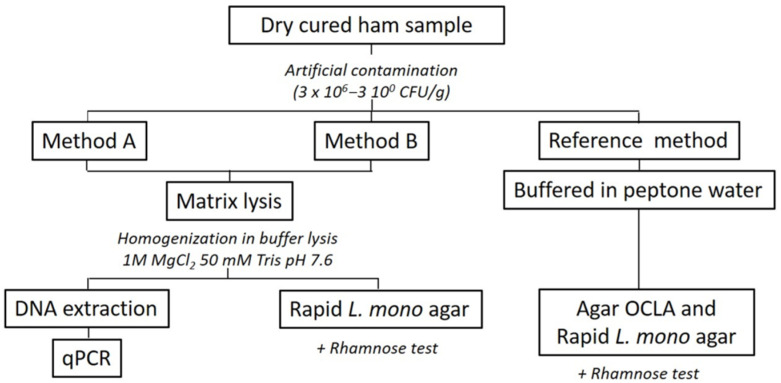
Scheme of the *L. monocytogenes* quantification methods steps.

Three different methods for the quantification of *L. monocytogenes* in meat products were evaluated (Figure 1). Two sets of 10 samples were evaluated in triplicate with each method (*n* = 180 samples). A scheme of the steps in each quantification procedure is represented in Figure 1.

##### Method A

Contaminated dry cured ham samples were first summited to a Matrix lysis, an adapted procedure previously described by Mester et al. [17] and Witte et al. [15]. According to this technique, 6 g of ham were mixed with 30 mL of lysis buffer (1 M MgCl_2_ y 50 mM Tris pH 7.6) and homogenized in a Stomacher bag (51 μm pore diameter) with a Stomacher 400 Circulator laboratory blender for 2 min at 260 rpm. The obtained liquid was transferred to a 50 mL sterile tubes and lysis buffer was added to a final total volume of 45 mL. Samples were incubated under shaking for 30 min at 37 °C. Then they were centrifuged at 3300× *g* at 30 °C. Supernatant was eliminated and the pellet was suspended in 45 mL of washing buffer (0.5% Tween 20 and 1X PBS) to be centrifuged 3300× *g* for 30 min at 30 °C. Supernatant was eliminated and the pellet suspended in 750 μL of PBS and centrifuged 9000× *g* for 5 min. This last step was performed twice. A DNA extraction was performed from the last obtained pellet according to the procedure previously described (Section 2.1) and the bacterial quantification was performed in triplicate for each sample by the optimized PCR method (*n* = 60). Besides, it was added in every well as an internal amplification control (IAC) (Exopol, Spain), composed by two primers and a probe bound to the fluorophore HEX that targeted the action gene.

A ham calibration curve was built from the analysis through this method. Three contaminated sample sets were analyzed through this method and the curve was built by confronting the obtained mean value of Cq obtained with qPCR versus the mean of bacteria culture concentration (log CFU/g) obtained with the reference method. Several parameters of the curve were determined according to Bustin et al. [20]; correlation coefficient (*R*^2^), linear dynamic range, efficiency, limits of detection (LOD), and quantification (LOQ), variation coefficient intra-assay and inter-assay (accepted <15%(European Pharmacopoeia, 2015), and analytical variability (standard deviation <0.25 log copies/reaction [21].

##### Method B

Matrix lysis was performed as in method A. The final pellet was suspended in 2.4 mL of PBS and colony count on Rapid L-mono agar in at 37 °C for 24 h (triplicate). The presumptive colonies were confirmed by the rhamnose test (37 °C for 24 h). This method B was performed to evaluate the matrix lysis effect on the final plate count compared it with the reference method. The presumptive colonies were confirmed by the rhamnose test (37 °C for 24 h).

##### Reference Method ISO 11290-2/A1:2018

In this procedure 6 g of the artificially contaminated ham were mixed with buffered peptone water (1/5 dilution) by homogenization with the Stomacher 400 Circulator laboratory blender for 2 min at 260 rpm. From each sample, tenfold dilutions were performed, and were inoculated on agar OCLA and chromogenic Rapid L. mono agar to be incubated at 37 °C/48 h and 37 °C/24 h, respectively (triplicate). The presumptive colonies were confirmed by the rhamnose test (37 °C for 24 h).

Once performed, the quantification of the experimentally contaminated samples through the three methods, the relative accuracy of methods A and B was determined as stablished in ISO 16140. It is a critical validation parameter that measures the closeness between the alternative method and the accepted reference value [20]. The relative accuracy was expressed as the percentage of CFU/g calculated by methods A and B versus the standard reference method. A relative of accuracy of 100% indicates a total agreement between the alternative and reference method.

### 2.3. Quantification of L. monocytogenes in Commercial Meat Products

There were selected a total of 43 samples of commercial meat products from Spain to evaluate the presence of *L. monocytogenes*: 38 samples of cured products (14 ham, 8 sausages, and 16 others), and 5 samples of deli meat.

First it was evaluated its presence in 25 g with the commercial iQ-Check *L. monocytogenes II* kit (Bio-Rad) PCR real time method. Then, the pathogen was quantified in those samples where it was detected.

From every sample 37 g were mixed in a sterile bag in the Stomacher Circulator 400 (260 rpm/2 min). Then the sample was divided in three; one portion of 25 g for detection purposes, and two portions of 6 g for quantifying. One portion of 6 g was evaluated with the optimized PCR method, while the other (6 g) was counted with the reference ISO 11290-2/A1:2018 method.

## 3. Results and Discussion

### 3.1. Optimization and Development of qPCR Method

For the validation of a PCR method for the quantification of *L. monocytogenes* the influence of annealing temperature, primers concentration, and probe concentration on the Cq and end-point were evaluated. Table 2 shows the Cq and end-point values obtained at the different experimental conditions tested.

Although the Cq and end-points values were similar despite the different conditions assayed, it was selected as an annealing temperature of 64 °C and 350 nM and 250 nM of primers and probe concentrations, respectively, which corresponds to the lowest Cq value, 30.31 ± 0.03, and the highest end-point, 0.34 ± 0.09.

### 3.2. qPCR Calibration Curves

Under the optimized experimental PCR conditions, a DNA calibration curve was built from the PCR analysis of serial DNA dilutions extracted from a pure culture. Equation (2)

Cq = −3.357 log fg DNA/reaction + 41.155; *R*^2^ = 0.999 (2)

DNA calibration curve slope was close to the optimum theoretical value −3.32, and the PCR reaction efficiency (E) was 98.5%, which is between the accepted range of 90–105% [20,22]. Both LOD and LOQ were 30 fg DNA/reaction or 9.5 genomic equivalents/PCR reaction since at this concentration all nine replicates were amplified and quantified. The obtained calibration curve results are similar to the ones reported in previous studies [19]. Quero et al. [21] obtained similar results in terms of dynamic linear range, correlation and efficiency, nevertheless obtained a LOD and LOQ of 1–5 genomic equivalent/PCR reaction. The DNA calibration curve results showed that the amplification parameters were adequate to perform the quantification of *L. monocytogenes.*

Then a cellular calibration curve was built from the triplicate PCR analysis of serial culture dilutions of a pure *L. monocytogenes* overnight solution Equation (3).

Cq = −3.503 log CFU/mL + 445.057; *R*^2^ = 0.998 (3)

The obtained curve presented six orders of magnitude linear dynamic range and the determined reaction efficiency within the accepted ranged, 92.9%. The LOD and LOQ values were 1.88 ± 0.10 log CFU/mL in both cases, which correspond to 2.4 ± 0.6 genomic equivalents of *L. monocytogenes*/reaction. These results are similar to other authors in terms of efficiency, dynamic linear range, and linearity [14,16]. Nevertheless, the LOD and LOQ obtain by Paul et al. [23] were 10 genomic equivalents/reaction, four times higher to the achieved in this study. The PCR quantification performed from serial dilution of a *L. monocytogenes* pure culture obtained in this is study provide a quantification curve with optimization values within the accepted ranges and lower concentration limits for the detection and quantification of the pathogen.

Finally, dry cured ham commercial samples were experimentally contaminated with a known bacterial concentration to be processed according to method A. After the matrix lysis and DNA extraction were performed as previously described in Section 2.2.2, *L. monocytogenes* was quantified under the PCR optimized conditions, in order to build a ham calibration curve (Equation (4)).

Cq = −3.313 log CFU/g of ham + 43.937; *R*^2^ = 0.987 (4)

The PCR method was capable of amplify from 6.82 ± 0.11 to 1.48 ± 0.10 log CFU/g within the accepted values of linearity, *R*^2^ = 0.9875, linear dynamic range, 5 orders of magnitude, and efficiency, E% = 100.4% [22]. The method LOD and LOQ was 1.48 ± 0.10 log CFU/g in both cases, which correspond to 30.1 ± 6.2 CFU/g or 6.0 ± 1.2 genomic equivalents of *L. monocytogenes*/reaction (Table 3).

Other authors have developed PCR methods for the detection and quantification of *L. monocytogenes* in different food matrices. Rantsiou et al. [14] developed a qPCR for meat products, cured ham among them, with values of correlation efficiency and lineal dynamic range within the accepted values. However, the LOD and LOQ was higher than the achieved in this work, 10^3^ CFU/g. Rodríguez-Lázaro et al. [24] quantification study results from pork and salmon samples were also limited in terms of dynamic rage (3 × 10^5^ to 3 × 10^7^ CFU/g) and LOD and LOQ (3 × 10^5^ CFU/g). Nevertheless, when they performed in another study a pre-PCR filtration treatment, the dynamic range improved to 4 logs, from 10^6^ to 10^3^ CFU/g [25].

The quantification via PCR of the microorganism in a solid food product is usually performed by a homogenization in buffered water causing the sample dilution 1:10. Then, an aliquot, generally 1 mL, is used for the DNA extraction and PCR quantification. The final DNA concentration from the microorganism is reduced five log unities regarding its initial concentration on the aliment [24]. Because of this, sample pretreatment before the qPCR has become an issue of great importance [18]. In order to develop a method with lower values of LOD and LOQ several authors have attempted the microorganism concentration before its analysis. Hough et al. [26] developed a qPCR method for *L. monocytogenes* in cabbage, with a centrifugation step for the concentration of the microorganism which extended the range at which the pathogen quantification to a final LOD of 1.4 × 10^2^ CFU/25 g. Besides, the sample concentration may cause the increase of sample inhibitors, affecting the DNA extraction or the PCR reaction. In order to reduce the presence of this inhibitors have also been reported experimental steps pre-PCR. D’Urso et al. [16] achieved a LOD of 10 CFU/10 g of yogurt when they performed centrifugation followed by a filtration before the DNA extraction. Rossmanith et al. developed a method based on matrix lysis for *L. monocytogenes* enrichment and the reduction of proteins, fatty acids, and carbohydrates achieving a LOD of 7.3 CFU/mL of milk. Finally, Mester et al. [17] and Witte et al. [15] applied a matrix lysis procedure for the pathogen quantification in dairy products and egg, reducing LOD and LOQ to <10 CFU/g.

**Figure 2:**
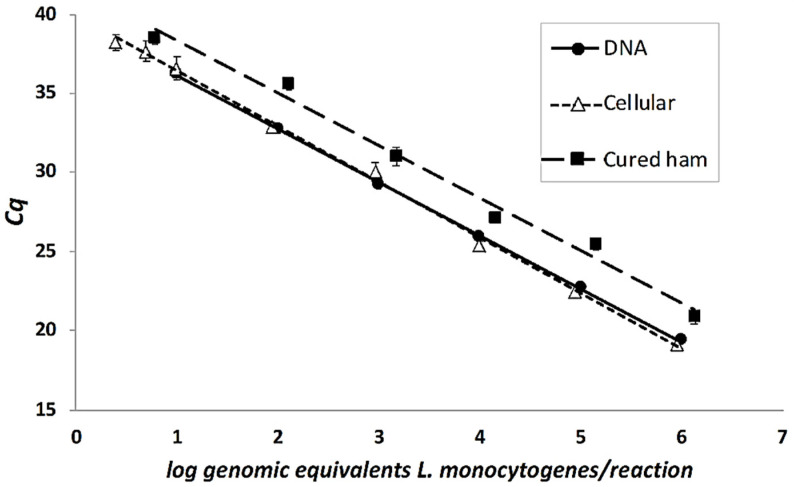
DNA, cellular, and cured ham calibration curves of *L. monocytogenes* ATCC 19114. Cq: quantification cycle.

As far as we know there are no other studies reporting limits of detection and quantification below the legal limit of 100 CFU/g for RTE meat products, which have a complex food matrix composition. In this work the method described by Witte et al. was applied [15], based in a matrix lysis solution, constituted by MgCl_2_ to solubilized proteins, and Tween 20 a non-ionic surfactant for fatty acids elimination. This pretreatment allowed the microorganism DNA extraction, without previous dilution, and the elimination of ham inhibitors, leading to LOD and LOQ values under legal limit (100 UFC/g or mL). As it can be observed Figure 2, where the three calibration curves built in this work are represented, dry cured ham curve is different to DNA and cellular curves, since its mean Cq was 2.6 higher than the other curves. The most probable cause is the aliment matrix effect, which has been reduced by the matrix lysis procedure. Quero et al. [21] reported a higher shift in the mean Cq regarding DNA and cellular *L. monocytogenes* in the study of cheese samples.

Even if they are preformed preliminary assays with pure DNA or pure *L. monocytogenes* strain, the qPCR final method should be further validated in the alimentary matrix in which the future control assays will be performed by the meat industry. Differences in the food composition and structure may affect to DNA extraction and amplification.

Method A repeatability and reproducibility were evaluated by the determination of intra and inter-assay variability. The following Table 3 shows the results of Cq of cured ham samples experimentally contaminated.

The variability determination was performed down to the lowest concentration at which all nine replicates were amplified, 1.48 ± 0.10 log CFU/g (6th level of contamination). Intra-assay CV and inter-assay CV varied between 0.06–2.81% and 0.84–1.91%, respectively, which is under the accepted maximum value 15%. Berrada et al. [27] on the development of a qPCR for *L. monocytogenes* in salads, obtained higher values of CV, 9–22%. In this study the quantification of this pathogen in cured ham was precise and reproducible from 6.82 ± 0.11 to 1.48 ± 0.10 log CFU/g. The analytical variability into this range of concentration was inferior to the maximum allowed limit of 0.25 log copies/reaction.

### 3.3. Comparison of Methods A and B versus Reference Method

The relative accuracy of the qPCR alternative method combined with matrix lysis was compared with the reference method ISO 11290-2. To do so, a total of 6 sets of 10 samples contaminated with tenfold dilutions of *L. monocytogenes* were analyzed with the three different procedures (2 sets for each method). The bacterial concentrations obtained and the relative accuracy of procedures A and B regarding the reference method are gathered in Table 4.

The loss of viable bacterial cells after the matrix lysis procedure was evaluated through the determination of the relative accuracy of method B regarding the reference method. This relative accuracy results varied into the range 92.70% to 108.69%, presenting higher values at lower concentrations. These results are higher than the obtained by Mester et al. [17], who used a similar matrix pretreatment in different food products (milk, dairy, and eggs). They reported an 88% of pathogen recovery after agar plate counting, which was considered satisfactory. An adequate sample preparation previous to the analysis is of great importance to avoid bacterial reduction by dilution Rossmanith et al., [18] and therefore underestimation of the pathogen concentration. In this work, the accuracy results reveal that there was not cell loss, so the sample preparation is adequate for the quantification of *L. monocytogenes* in this alimentary matrix.

The quantification with qPCR after the matrix lysis procedure was also compared with the reference method and the results of relative accuracy are reported in Table 5. The values obtained were comprised among 95.83% and 105.20%. Despite the complexity of meat matrix, these results are into a narrow interval and close to 100%, which reveal a high concordance with the reference method in the quantification of this pathogen. The relative accuracy results obtained were better than the previously reported by [24,25] (89.12%–116.33%) in meat products. Nevertheless, D’Urso et al. [16], who studied *L. monocytogenes* in milk, dairy products, and eggs, also reported quantitative PCR results with an excellent correspondence with the reference method (95.72–104.03%).

The qPCR method has adjusted to all quantification parameters required and it can also be performed in a shorter time than the reference method. The complete procedure matrix lysis + DNA extraction + 45 PCR cycles takes approximately 2 h while the reference method requires at least 4 days, including the incubation periods and the biochemical identification.

### 3.4. L. monocytogenes Quantification Meat Commercial Products

A total of 43 commercial samples were analyzed to evaluate the presence of *L. monocytogenes* in 25 g and it was detected in 23.3% of the samples: 10 of dry cured ham and other cured products (Table 5). The methods of reference and qPCR were applied to perform the quantification of the microorganism in those that resulted positive. Other authors have also reported the low counting for this kind of product. Gómez et al. detected the pathogen in 27.9% of the analyzed meat samples but the quantification with the reference method was in most of them under 100 CFU/g. Thévenot et al. [28] detected the pathogen in 10% of the samples being the final counting <3 CFU/g in all of them with the reference method. Rodriguez-Lazaro et al. [25] observed higher presence in chorizo than in other meat products detecting the microorganism in 77% of them, although the quantification revealed that *L. monocytogenes* concentration was <10 CFU/g.

In this work, after the quantification through the reference method, microbial growth was not observed in the chromogenic agar, so the presence of *L. monocytogenes* in all positive samples was <5 CFU/g. When the qPCR method was applied, 9 of the samples presented a concentration under the LOQ (<30.1 ± 6.2 CFU/g), while 1 of them presented a mean Cq of 37.84 ± 0.28, which correspond according to the ham calibration curve to 1.84 ± 0.08 log CFU/g or 69.1 ± 13.9 CFU/g. Nevertheless, this result is under the legal limit of 100 CFU/g, stablished by the (Commission Regulation (EC) No 2073/2005, 2005).

These differences in quantification among the qPCR method and the reference method, have already been reported in other food products. Mester et al. [17] observed significant differences when quantifying positive samples of cheese for *L. monocytogenes*. Furthermore, for experimentally samples contaminated with 100 CFU/g the reference method quantified between 13–741 CFU/g and 15–646 CFU/g [10].

As it has been previously observed, the reference method might be insufficiently sensitive to quantify the microorganism in those samples with a low concentration because of food matrix effect or concomitant microbial flora. Nevertheless, the presence of stressed bacteria with difficulty to grow in selective medium could affect negatively to the quantification reference method underestimating the pathogen concentration [29].

## 4. Conclusions

In conclusion, the qPCR method for quantifying *L. monocytogenes* complies with all assayed validation parameters. The pre-treatment with matrix lysis permitted the dilution of the PCR inhibitors and thereby significantly reduced the limits of detection and quantification, making this work the first to reports a procedure to quantify *L. monocytogenes* in meat products with an LOQ as low as 30 CFU/g.

Because of the observed insufficient precision of the reference method at low concentrations, this qPCR procedure is presented to the food industry as an excellent alternative to quantify this pathogen in meat samples. This substitution allows a faster and more sensitive quantification of the microorganism in multiple samples simultaneously, that can be included in the HACCP system.

## Figures and Tables

**Table 1:** Primers and probe sequence used in this study.

Primers/Probe	Sequence (5′–3′)	Amplicon (bp)
Forward	5′-TGCAAGTCCTAAGACGCCA-3′	113
Reverse	5′-CACTGCATCTCCGTGGTATACTAA-3′	
Taqman probe	FAM-5′CGATTTCATCCGCGTGTTTCTTTTCG-BkFQ	

**Table 2:** Cq (quantification cycle) and end-point values ± their standard deviation at different annealing temperatures and primers/probe concentrations.

Annealing (°C)	350/250 ^1^ (nM)		350/125 ^1^ (nM)		250/250 ^1^ (nM)		250/125 ^1^ (nM)	
	Cq ^2^	End-Point ^3^	Cq ^2^	End-Point ^3^	Cq ^2^	End-Point ^3^	Cq ^2^	End-Point ^3^
65	31.18 ± 0.05	0.21 ± 0.02	32.91 ± 0.28	0.07 ± 0.01	32.50 ± 0.13	0.15 ± 0.00	32.48 ± 0.55	0.12 ± 0.03
64.7	30.77 ± 0.02	0.27 ± 0.01	32.17 ± 0.24	0.10 ± 0.01	31.88 ± 0.40	0.20 ± 0.02	31.87 ± 0.10	0.15 ± 0.01
64	**30.31 ± 0.03**	**0.34 ± 0.09**	31.59 ± 0.05	0.13 ± 0.01	30.81 ± 0.52	0.27 ± 0.08	31.07 ± 0.29	0.19 ± 0.03
63.1	31.56 ± 0.42	0.16 ± 0.04	31.97 ± 0.05	0.10 ± 0.00	31.99 ± 0.69	0.13 ± 0.08	31.27 ± 0.03	0.17 ± 0.00
62	30.59 ± 0.45	0.33 ± 0.10	32.04 ± 0.29	0.11 ± 0.01	30.75 ± 0.57	0.26 ± 0.09	31.09 ± 0.01	0.20 ± 0.01
61.1	30.89 ± 0.20	0.31 ± 0.00	31.88 ± 0.06	0.13 ± 0.00	31.06 ± 0.12	0.25 ± 0.02	31.19 ± 0.01	0.19 ± 0.01
60.4	31.05 ± 0.00	0.30 ± 0.02	32.16 ± 0.04	0.12 ± 0.00	31.18 ± 0.13	0.23 ± 0.02	31.43 ± 0.07	0.18 ± 0.01
60	31.87 ± 0.24	0.18 ± 0.03	32.77 ± 0.88	0.09 ± 0.04	32.08 ± 0.47	0.14 ± 0.02	31.90 ± 0.79	0.15 ± 0.07

^1^ Concentration of primers and probe used in qPCR (nM); ^2^ Mean Cq ± standard deviation of three replicates; ^3^ Mean *Endpoint* medio; ± standard deviation of three replicates; Bolded numbers are the best results.

**Table 3:** PCR validation results of experimentally contaminated cured ham samples.

Level ^1^	Ref Method ^2^	qPCR Method							
	log CFU/g	Cq ^3^	Ratio ^4^	Intra-Assay CV ^5^			Inter-Assay CV ^6^	Variability ^7^	Max Variability ^8^
1	6.82 ± 0.11	20.85 ± 0.39	9/9	0.43	0.42	2.81	1.91	0.12	1.57
2	5.85 ± 0.03	25.49 ± 0.39	9/9	0.90	0.25	2.28	1.54	0.16	1.22
3	4.86 ± 0.05	27.12 ± 0.22	9/9	0.89	1.03	0.61	0.84	0.07	1.09
4	3.87 ± 0.06	30.98 ± 0.58	9/9	0.06	1.79	0.46	1.88	0.18	0.80
5	2.80 ± 0.03	35.56 ± 0.39	9/9	0.82	0.97	1.08	1.12	0.12	0.46
6	1.48 ± 0.10	38.35 ± 0.56	9/9	0.94	2.49	0.98	1.48	0.17	0.24

^1^ Level of contamination assayed; ^2^ Mean ± standard deviation of the replicate analysis through the reference method; ^3^ Mean ± standard deviation of the obtained Cq from 9 replicates; ^4^ Ratio: positive PCR/total PCR; ^5^ Intra-assay CV of the log CFU/g determined with PCR expressed in percentage; ^6^ Inter-assay CV of the log CFU/g determined with PCR expressed in percentage; ^7^ standard deviation of log gen copies hlyA/PCR reaction; ^8^ Maximum standard deviation value that can be achieved in each contamination level, determines as 0.25 log gen hlyA copies/reaction.

**Table 4:** Bacterial concentration (±sd) and relative accuracy (±sd) of methods A and B regarding ISO 11290-2 for the quantification of *L. monocytogenes* in cured ham samples.

	Ref Method	Method A		Method B	
Level	Plate Counting (log CFU/g)	qPCR (log CFU/g)	Relative Accuracy (%)	Plate Counting (log CFU/g)	Relative Accuracy (%)
1	6.41 ± 0.38	6.38 ± 0.42	99.49 ± 0.92	6.54 ± 0.49	102.03 ± 2.26
2	5.38 ± 0.25	5.51 ± 0.19	102.47 ± 1.74	5.45 ± 0.42	101.30 ± 4.39
3	4.36 ± 0.45	4.31 ± 0.29	99.22 ± 5.08	4.70 ± 0.15	92.70 ± 6.98
4	3.15 ± 0.17	3.02 ± 0.19	95.83 ± 1.22	3.21 ± 0.18	101.90 ± 0.30
5	2.15 ± 0.09	2.13 ± 0.07	99.11 ± 1.26	2.23 ± 0.13	103.72 ± 2.41
6	1.80 ± 0.11	1.89 ± 0.09	105.02 ± 2.02	1.93 ± 0.11	97.80 ± 0.62

**Table 5:** Detection and quantification of *L. monocytogenes* in different commercial meat products.

		Detection IQ-Check	Quantification	
	n	qPCR Detected/25 g	qPCR (CFU/g)	ISO 11290-2 (CFU/g)
Cured				
- Ham	14	4	<30.1 ± 6.2 (*n* = 4)	<5
- Sausage	8	2	<30.1 ± 6.2 (*n* = 2)	<5
- Others	16	4	<30.1 ± 6.2 (*n* = 3) 69.1 ± 13.9 (*n* = 1)	<5
Deli meat	5	*nd*	*na*	*na*
Total	43	10		

nd: not detected; na: not analyzed.
